# ZNRD1 and Its Antisense Long Noncoding RNA ZNRD1-AS1 Are Oppositely Regulated by Cold Atmospheric Plasma in Breast Cancer Cells

**DOI:** 10.1155/2020/9490567

**Published:** 2020-05-05

**Authors:** Hyeon Woo Kim, Dawoon Jeong, Juyeon Ham, Heejoo Kim, Hwee Won Ji, Eun Ha Choi, Sun Jung Kim

**Affiliations:** ^1^Department of Life Science, Dongguk University-Seoul, Goyang 10326, Republic of Korea; ^2^Plasma Bioscience Research Center, Kwangwoon University, Seoul 01897, Republic of Korea

## Abstract

Cold atmospheric plasma (CAP) has been recognized as a potential alternative or supplementary cancer treatment tool, which is attributed by its selective antiproliferation effect on cancer cells over normal cells. Standardization of the CAP treatment in terms of biological outputs such as cell growth inhibition and gene expression change is essential for its clinical application. This study aims at identifying genes that show consistent expression profiles at a specific CAP condition, which could be used to monitor whether CAP is an appropriate treatment to biological targets. To do this, genes showing differential expression by two different CAP treatment conditions were screened in the MCF-7 breast cancer cells. As a result, ZNRD1 was identified as a potential marker with being consistently upregulated by 600 s but downregulated by the 10 × 30 s CAP treatment scheme. Expression of ZNRD1 was increased in breast cancer tissues compared to normal tissues, judged by cancer tissue database analysis, and supported by the antiproliferation after siRNA-induced downregulation in MCF-7. Interestingly, the antisense long noncoding RNA (lncRNA) of ZNRD1, ZNRD1-AS1, was regulated to the opposite direction of ZNRD1 by CAP. The siRNA-based qPCR analysis indicates that ZNRD1 downregulates ZNRD1-AS1, but not *vice versa*. ZNRD1-AS1 was shown to increase a few cis-genes such as HLA-A, HCG9, and PPP1R11 that were also regulated by CAP. Altogether, this study identified a pair of gene and its antisense lncRNA of which expression is precisely controlled by CAP in a dose-dependent manner. These genes could help elucidate the molecular mechanism how CAP regulates lncRNAs in cancer cells.

## 1. Introduction

Cold atmospheric plasma (CAP) is a specific type of plasma produced at low atmospheric temperature. CAP consists of charged particles, free radicals, neutral atoms, ultraviolet (UV) photons, and reactive oxygen and nitrogen species [1, 2]. When CAP is applied to biological materials ranging from cultured cells to xenografted tumor tissues, it successfully induces cell death [3–5]. Above all, CAP has the advantage of preferentially damaging cancer cells over normal cells. This is attributed to the higher ROS level in cancer cells than in normal cells [6]. CAP increases the ROS level in both cell types, but the resulting ROS level in cancer cells is past the threshold of cellular survival, leading to cell death, while still below the threshold in normal cells [7, 8]. This characteristic of CAP has been utilized in various cancer cell types for cancer treatment in vitro cultures cells and in vivo animal models [9–11].

In the course of using CAP as a medical treatment option, one of the pivotal considerations is to standardize the whole process from the plasma-generating apparatus through the composition of medium to the response of target cells. The plasma sources are relatively well established for standardization [12, 13]. Currently, two types of devices have been developed: DBD and jet type [14]. In both types, the treatment conditions can be represented using V and Hz with time set for the required duration. Park et al. applied 0.46 kV and 12.89 kHz for 600 s, which caused MCF-7 breast cancer cell apoptosis of up to 13.5% [3]. In another study, plasma treatment of 20 kV and 500 Hz for 120 s induced Jurkat leukemia cancer cell apoptosis of up to 26.6% [15]. The response of cells to plasma is also affected by the composition of media added to the cultured cells [16–18]. The need for standardization of media is more essential in the case of using CAP indirectly, via plasma-treated medium (PTM). In this case, the concentration of specific chemicals dissolved in the media is determined. Hattori et al. used PTM to treat Capan-2 pancreatic cancer cells, inducing 47% cancer cells apoptosis [19].

The top priority of standardization is the outcome of cellular and/or molecular change induced by CAP. The efficacy of CAP can be expressed with the percentage of cells that are induced to death. However, standardization is not easy, because the death rate is vulnerable depending on the origin of cultured cells and culture conditions [20, 21]. As the molecular change, including RNA and protein expression, is the eventual response by CAP, searching for marker genes that show expression change in proportion to the CAP energy will contribute to establishing the standardization of CAP treatment. To date, various genes have been identified as having expression affected by plasma. Among them, DNA damage- and apoptotic pathway-related genes such as *γ*-H2AX [22] and caspases [10, 23] have been frequently identified. In spite of the large number of affected genes, few studies have shown the association between the gene expression level and the CAP treatment condition, which is an essential requisite to establish marker genes.

In this study, we identified ZNRD1 and its antisense long noncoding RNA (lncRNA) ZNRD1-AS1, the expression of which was increased or decreased in two different CAP treatment conditions. In addition, the regulatory relationship of the two genes was elucidated through inhibition study of each gene. These genes, to the best of our knowledge, are the first pair of a coding gene and antisense lncRNA to show opposite expression by different CAP energies.

## 2. Materials and Methods

### 2.1. Cell Culture and CAP Treatment

Human breast cancer cell lines MCF-7 and T-47D and a normal cell line, MCF-10A, were purchased from the American Type Culture Collection (ATCC). Cancer cell lines were cultured in RPMI1640 (Gibco, Grand Island, NY, USA) supplemented with 10% fetal bovine serum and 2% penicillin and streptomycin. MCF-10A was cultured in MEGM (Lonza, Basel, Switzerland) supplemented with the MEGM SingleQuot Kit and 100 ng/mL cholera toxin. All cells were incubated in a humidified cell incubator with 5% CO_2_ at 37°C. The mesh–dielectric barrier discharge- (DBD-) type CAP device was developed at the Plasma Bioscience Research Center of Kwangwoon University (Seoul, Korea) (Figure S1) [24]. The effect of CAP on the production of reactive oxygen or nitrogen species was examined in our previous study [25]. The voltage, current, and frequency of the CAP were 0.38 kV, 12.6 mA, and 12.9 kHz, respectively (Table S1). CAP was generated with 1 L/min argon gas and exposed 10 times for 30 s every hour or in single treatment for 600 s to the cells at a 4 mm distance from the surface of the medium.

### 2.2. Cell Transfection

siRNAs for ZNRD1 and ZNRD1-AS1 were synthesized by Bioneer (Daejeon, Korea) and Qiagen (Redwood City, CA, USA), respectively. A control siRNA (siNC) was synthesized by Bioneer. Cells were seeded in 60 mm plates and transfected at a final concentration of 20 or 40 nM using Lipofectamine RNAi MAX (Invitrogen, Carlsbad, CA, USA) in serum-free Opti-MEM I Medium (Gibco) according to the manufacturer's protocol. RNA extraction and functional assay were performed 24 h after transfection. The sequence of siRNAs is shown in Table S2.

### 2.3. Colony Formation Assay

For colony formation assay, 5 × 10^3^ cells were seeded in 60 mm plates with 2 mL medium. CAP treatment was performed 24 h after transfection and the cells were maintained for 14 days. The colonies were gently washed with PBS, fixed with methanol/acetic acid (7 : 1), and then stained with 0.2% crystal violet (Sigma-Aldrich, St. Louis, MO, USA). The relative colony area was analyzed using the ImageJ software [26].

### 2.4. Cell Proliferation Assay

The cells were seeded in a 96-well plate at a density of 2 × 10^3^ cells/well and transfected with siRNAs. CAP treatment was performed 24 h after transfection, and the cell growth rate was monitored at 0, 24, 48, 96, and 144 h. At each time point, 10 *μ*L of CCK-8 solution (Dojindo, Kumamoto, Japan) was added to each well, and the absorbance was measured after 1 h using a microplate reader at 450 nm.

### 2.5. Quantitative Real-Time RT-PCR (qPCR)

Total RNA was isolated using the AllPrep DNA/RNA/miRNA Universal Kit (Qiagen) with a 50 *μ*L elution volume according to the manufacturer's protocol. cDNA was synthesized from 2 *μ*g of RNA using the ReverTra Ace qPCR RT Master Mix with gDNA remover (Toyobo, Osaka, Japan). qPCR was performed using a KAPA SYBR Fast qPCR Kit (Kapa Biosystems, Woburn, MA, USA) on the ABI 7300 instrument (Applied Biosystems, Foster City, CA, USA). GAPDH was used for normalization and calculated using the 2^-*ΔΔ*Ct^ method. Primer sequences used for qPCR are listed in Table S2.

### 2.6. Methylation-Specific Polymerase Chain Reaction (MSP)

Chromosomal DNA was extracted using the AllPrep DNA/RNA/miRNA Universal Kit (Qiagen) with a 50 *μ*L elution volume. Bisulfite conversion was conducted using 1 *μ*g of DNA with an EZ DNA methylation Kit (Zymo Research, Irvine, CA, USA) on the ABI 7300 instrument (Applied Biosystems). A methylation index (*β*) was calculated for each sample using the following formula: methylation index = 1/[1 + 2^−(CTu − CTme)^] × 100%. CTu is the average cycle threshold (CT) obtained from PCR analysis using the unmethylated primer pair, and CTme is average CT obtained using the methylated primer pair. Primer sequences used for MSP are listed in Table S2.

### 2.7. Statistical Analysis

The methylation data for the CpG at the ZNRD1 promoter in breast cancer patients was retrieved from the TCGA Wanderer database (http://www.maplab.imppc.org/wanderer). The expression data for ZNRD1 and ZNRD1-AS1 was retrieved from the GEPIA database (http://gepia.cancer-pku.cn). All experimental results were independently performed at least three times and analyzed by the two-sided Student's *t*-test. Differences were considered statistically significant when the *P* value was lower than 0.05.

## 3. Results

### 3.1. ZNRD1 and ZNRD1-AS1 Are Oppositely Regulated by CAP

**Figure 1:**
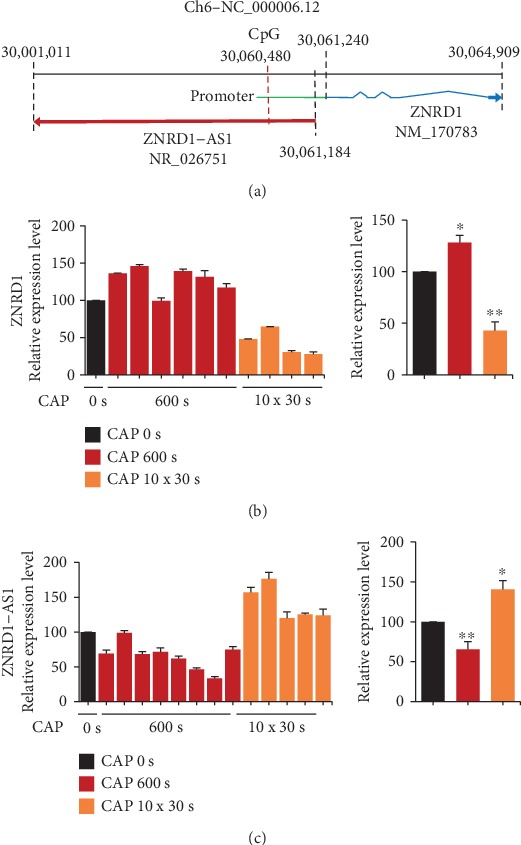
Induction of opposite expression of ZNRD1 and ZNRD1-AS1 under different CAP conditions. (a) Schematic structure of ZNRD1 and ZNRD1-AS1 on chromosome. ZNRD1 is indicated by the blue line, with its promoter in green. ZNRD1-AS1 is indicated by the red line. The arrowheads are the transcription direction of each gene. The location of the CpG hypermethylated by CAP is indicated with a red vertical dotted line. (b, c) Expression of ZNRD1 and7 ZNRD1-AS1 was examined by qPCR after treatment of MCF-7- cells with CAP at the indicated doses. The CAP treatment was performed multiple times, with each time in triple culture dishes. The overall average is indicated on the right with mean ± SE. ^∗^*P* < 0.05, ^∗∗^*P* < 0.01.

In our previous genome-wide methylation analysis, a specific CpG site near the ZNRD1 promoter (-760 from the transcription start site) was identified to be hypermethylated (Δ*β* = 0.198, fold change = 2.152) by CAP in the MCF-7 breast cancer cells [3]. This study was performed to elucidate the mechanism by which CAP regulates the methylation level of CpG and ZNRD1 expression. At first, the CpG was mapped on the chromosome, found to be located 760 bases upstream of the transcription start site of ZNRD1 (Figure 1(a)). Notably, an antisense lncRNA, ZNRD1-AS1, is encoded from the other strand of ZNRD1 with sharing the CpG at its transcript-coding region. ZNRD1 is a zinc ribbon domain-containing protein and is downregulated in a few cancers including esophageal cancer [27] and gastric cancer [28]. ZNRD1-AS1 is the antisense lncRNA of ZNRD1 and is located in the upstream region of the ZNRD1 [29]. Little is known about the function of the lncRNA in the development of cancer, and none is available in breast cancer.

The expression of ZNRD1 after CAP treatment was examined by qPCR. The MCF-7 cells were independently treated six times by CAP. Results showed that ZNRD1 was upregulated by CAP of 600 s, being confirmed in five of six independent experiments (Figure 1(b)). A different CAP energy of 30 s for 10 times with an hour interval was also applied to the cell. Surprisingly, expression of ZNRD1 was rather decreased in the case of the 10 × 30 s treatment, confirmed by four independent experiments (Figure 1(b)). ZNRD1-AS1 also showed an opposite regulation under the two CAP treatment schemes, but surprisingly showing the opposite expression pattern to that of ZNRD1 (Figure 1(c)). The treatment of argon gas only did not induce any significant change of gene expression (Figure S2).

To see any association between the methylation of the CpG and expression of the two genes, the methylation level of the CpG was examined after treatment of MCF-7 with CAP. Results showed that both 600 s and 10 × 30 s CAP induced hypermethylation, although the increased methylation levels were different, with 96.2% increase in 600 s and 38.4% increase in 10 × 30 s (Figure S3). This result indicates that the CpG site does not affect the expression of ZNRD1 and ZNRD1-AS1, although its methylation level is influenced by CAP.

### 3.2. ZNRD1 Induces Downregulation of ZNRD1-AS1 with Being Upregulated in Breast Cancer

**Figure 2:**
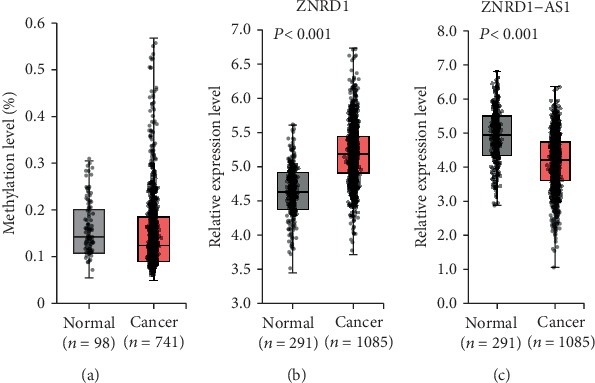
DNA methylation and expression profile of ZNRD1 and ZNRD1-AS1 in breast cancer. (a) The methylation level of the specific CpG (CpG ID; cg02078039) at ZNRD1 promoter in breast cancer tissues, which was hypermethylated by CAP in MCF-7, was examined using the data retrieved from the TCGA Wanderer database. No significant difference was found between the normal and cancer tissues. Expression of ZNRD1 (b) and ZNRD1-AS1 (c) was analyzed for tissues in the database GEPIA. Upregulation of ZNRD1 and downregulation of ZNRD1-AS1 were observed in breast cancer tissues. *n*: sample number.

The CpG methylation and expression of ZNRD1 and ZNRD1-AS1 were analyzed from the data of normal and cancer tissues, of which information was retrieved at the TCGA Wanderer database and GEPIA database. The methylation level of the CpG did not show a significant difference between the normal breast tissues (*n* = 98) and cancer tissues (*n* = 741) (Figure 2(a)). The expression of ZNRD1 was upregulated in the cancer tissues (*n* = 1,085) compared to the normal tissues (*n* = 291) (*P* < 0.001) (Figure 2(b)). Meanwhile, expression of ZNRD1-AS1 was downregulated in the cancer tissues compared to the normal tissues (Figure 2(c)). These results are in parallel with those of the CAP-treated MCF-7 cells, i.e., opposite regulation of the two genes by CAP, but no association with CpG methylation.

**Figure 3:**
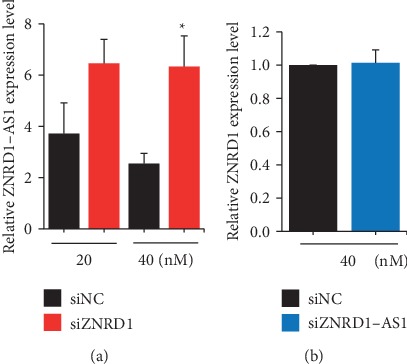
ZNRD1 downregulates ZNRD1-AS1 in the MCF-7 cells. Downregulation of ZNRD1 or ZNRD1-AS1 was induced in the MCF-7 cells using siRNA, and their expression was examined by qPCR. (a) Expression of ZNRD1-AS1 after transient transfection of siRNA for ZNRD1 (siZNRD1). (b) Expression of ZNRD1 after transient transfection of siRNA for ZNRD1-AS1 (siZNRD1-AS1). All of the experiments were performed in triplicate, and the values are presented as the mean ± SE. ^∗^*P* < 0.05.

To examine whether the opposite expression of ZNRD1 and ZNRD1-AS1 by CAP is due to the regulation by each other, expression of each gene was examined after inhibiting expression of the other using siRNA (Figure S4). A siRNA targeting ZNRD1 induced upregulation of ZNRD1-AS1; however, siRNA targeting ZNRD1-AS1 did not affect the expression of ZNRD1 (Figure 3). This result implies that ZNRD1 is upregulated and downregulated by CAP of 600 s and 10 × 30 s, respectively, and the altered expression accompanies the down- and upregulation of ZNRD1-AS1.

### 3.3. ZNRD1 Stimulates Proliferation of MCF-7 Breast Cancer Cells

**Figure 4:**
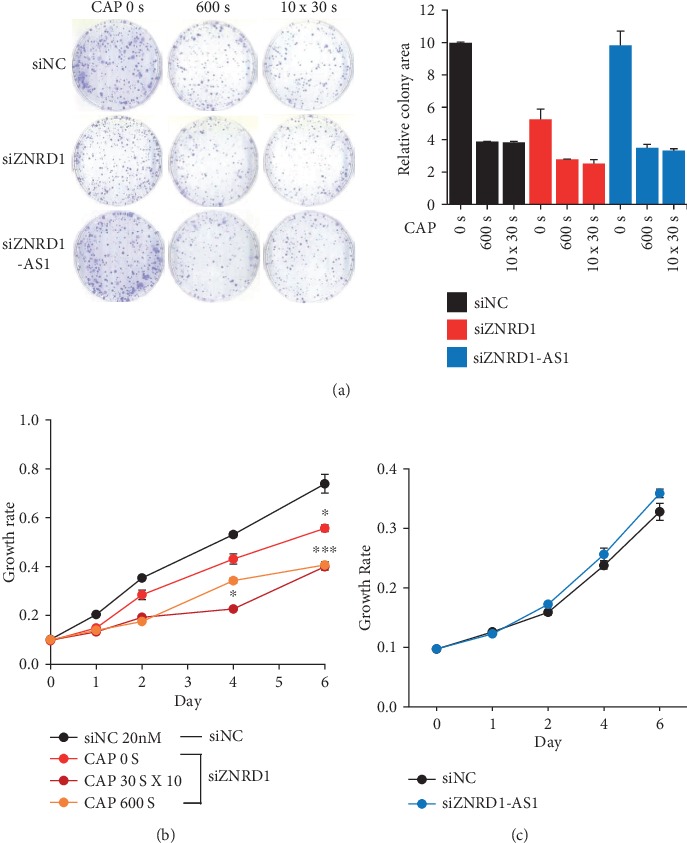
ZNRD1 but not ZNRD1-AS1 stimulates proliferation of MCF-7. (a) Either ZNRD1 or ZNRD1-AS1 was downregulated in MCF-7 using siRNA, and cell survival was examined by colony formation assay. Three independent experiments were performed, and representative images are shown. The effect of the siRNA-driven downregulation of ZNRD1 (b) or ZNRD1-AS1 (c) on cell proliferation was examined by CCK-8 assays. The experiments were performed independently at least three times, and the values are presented as the mean ± SE. siNC: control siRNA; siZNRD1: siRNA for ZNRD1; siZNRD1-AS1: siRNA for ZNRD1-AS1. ^∗^*P* < 0.05, ^∗∗∗^*P* < 0.001.

Although ZNRD1 and ZNRD1-AS1 were revealed to contribute the development of cancer in a few cancer types, their role in breast cancer remains obscure. To address their contribution to the proliferation of breast cancer cells, each gene was downregulated in MCF-7 using siRNA and cell proliferation was monitored by colony formation assay and a dye-based growth rate assay. As a result, when ZNRD1 was suppressed, less colony formation was observed with being further inhibited by combined CAP treatment (Figure 4(a)). The dye-based growth rate assay also showed the similar inhibition pattern (Figure 4(b)). However, when ZNRD1-AS1 was suppressed, no significant change of cell proliferation was found in either the colony formation assay or dye-based growth rate assay (Figure 4(a) and 4(c)).

**Figure 5:**
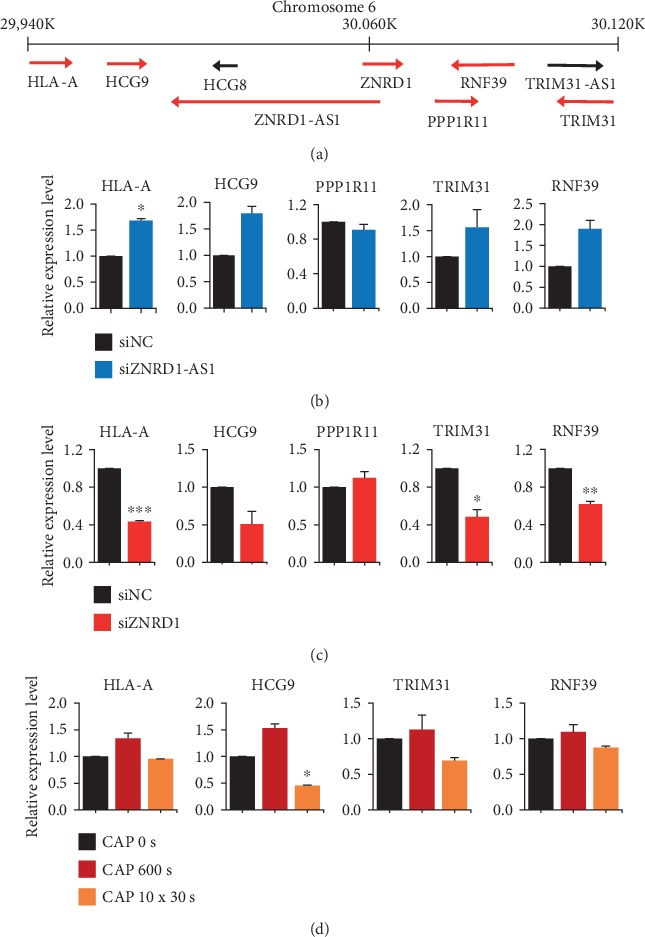
The effect of siRNA for ZNRD1-AS1 and CAP on the regulation of cis-genes. (a) A schematic map of the relative position of ZNRD1-AS1 and its nearby cis-genes. The numbers on the horizontal line are the nucleotides of a subfragment on chromosome 6. The arrows indicate the expression direction. The MCF-7 cells were treated with a siRNA for ZNRD1-AS1 (b) or a siRNA for ZNRD1 (c), and the expression levels of the cis-genes were examined using qPCR. (d) The MCF-7 cells were treated with CAP and the expression of the cis-genes was examined using qPCR. All of the experiments were performed independently at least three times, and the values are presented as the mean ± SE. ^∗^*P* < 0.05, ^∗∗^*P* < 0.01, ^∗∗∗^*P* < 0.001.

Many lncRNAs have been known to regulate nearby genes in the so-called cis-mode. To identify any cis-genes regulated by ZNRD1-AS1, expressions of five cis-genes were examined by qPCR after suppressing ZNRD1-AS1 using siRNA (Figure 5(a)). As a result, four genes were upregulated while one gene was slightly downregulated (Figure 5(b)). Notably, expression of the cis-genes was reversed when ZNRD1 was suppressed by siRNA, supporting our observation that ZNRD1-AS1 is downregulated by ZNRD1 (Figure 5(c)). Furthermore, CAP treatment for 600 s and 10 × 30 s induced upregulation and downregulation of the four genes, respectively, which showed upregulation when siRNA for ZNRD1-AS1 was treated, although only HCG9 showed statistical significance (Figure 5(d)).

## 4. Discussion

This study was performed to identify genes that show consistent expression change under independent CAP treatments and thereby to be able to monitor whether CAP is an appropriate treatment to biological targets. Two different CAP treatment conditions induced the opposite expression for ZNRD1 and ZNRD1-AS1. However, the specific CpG at the ZNRD1 promoter (Figure 1) was hypermethylated by the two different CAP treatment conditions in MCF-7 cells. Furthermore, no significant difference of methylation level between normal and cancer tissues in breast was found (Figure 2). These facts imply that the specific CpG is not responsible for the regulation of ZNRD1 and ZNRD1-AS1. Other CpG(s) or regulatory mechanism than the epigenetic way may be responsible for the regulation of the genes.

Considering the fact that ZNRD1 is oncogenic in breast cancer, the CAP treatment condition of 10 × 30 s is recommended to inhibit the MCF-7 cancer cell growth, because CAP in that condition, rather than 600 s, suppressed ZNRD1. In accordance, a few cis-genes of ZNRD1-AS1, HLA-A, HCG9, TRIM31, and RNF39 were upregulated at 600 s, but downregulated at the 10 × 30 s CAP treatment. Among the four genes, HLA-A and TRIM31 are known for their association with cancer, but their contribution to cancer development is not the same. Downregulation of HLA-A expression has been known to contribute to a poor prognosis in cancer patients, suggesting its tumor-suppressive activity [30]. TRIM31 is an oncogene promoting proliferation, invasion, and migration of glioma cells through Akt and NF-*κ*B pathways [31, 32]. More accumulation of data is needed to comprehensively understand the contribution of the cis-genes of ZNRD1-AS1 to the tumor development.

A limitation remains for the CAP treatment scheme because the cell growth inhibition appeared at the both CAP conditions. Therefore, it is speculated that just alteration of ZNRD1 and ZNRD1-AS1 is not sufficient to induce cancer cell death. Setting up CAP conditions that include one inhibiting cancer cell growth and another stimulating cell growth would be helpful to establish more reliable marker genes. This seems possible, as low dose of CAP stimulated cancer cell growth in a few cancers, even though CAP induced cancer cell death in the majority of previous studies [33]. Low doses of CAP activated fibroblast proliferation in wound tissue of mouse model, but over doses suppressed wound healing by causing cell death [33]. Another limitation of this work is in the lack of genome-wide expression analysis for the different CAP treatments. ZNRD1 and ZNRD1-AS1 were just selected from a genome-wide methylation array dataset. To further screen marker genes, extensive analysis through a genome-wide approach after treatment of CAP at diverse CAP conditions is required.

For the standardization of CAP, CAP condition, chemical composition of cell culture, media, and cellular responses are key factors, but standardizing these alone is still insufficient. For example, the distance from the outlet of CAP device to the surface of culture media should be also considered. In addition, different cancer cell types represent their unique molecular response. In our previous study, even two cell lines originated from breast tissue, MCF-7 and MDA-MB-231, showed a genome-wide difference of DNA methylation by the same CAP treatment condition [3]. In addition, MCF-10A cells showed the similar expression profile for ZNRD1 and ZNRD1-AS1 by CAP, but T-47D showed the opposite expression at the two CAP conditions in the current study (Figure S5). Nonetheless, the expression profile that ZNRD1-AS1 is downregulated when ZNRD1 is upregulated, and vice versa, has not changed even in T-47D. The standardization becomes further complicated when even a single cell line shows various responses depending with the genetic and physiological status, such as number of subcultures. Therefore, a comprehensive approach is essential for the development of reliable marker genes. Another finding of this study is to have established the regulatory relationship between ZNRD1 and ZNRD1-AS1. A few studies dealt with the expression association between the two genes in cancer, all presenting their opposite expression profile [29, 34]. However, no regulatory pathway has been identified. Our current study indicates that ZNRD1 downregulates ZNRD1-AS1 with no feedback regulation.

## 5. Conclusions

ZNRD1 and its antisense lncRNA ZNRD1-AS1 were revealed to be regulated in opposite ways depending on the CAP treatment conditions. The specific condition of 10 × 30 s was found to suppress the ZNRD1 expression, while the 600 s scheme induced upregulation. A regulatory pathway that CAP regulates ZNRD1, which in turn downregulates ZNRD1-AS1, is suggested. In addition, a few cis-genes of ZNRD1-AS1 were found to be regulated by the lncRNA and CAP. The two genes could contribute to precisely establishing the relationship between the CAP treatment condition and target gene expression.

## Data Availability

All generated and analyzed data used to support the findings of this study are included within the article.

## Supplementary Materials

Supplementary Materials Table S1: specification and electrical characterization of the 90 mm mesh DBD plasma device. Table S2: sequences of primers for qPCR and siRNAs employed in this study. Figure S1: CAP source used in this study. Schematic diagram (a) and voltage-current waveform (b) of the 90 mm mesh DBD plasma device are shown. (c) pH change of culture media after CAP treatment. Figure S2: treatment of argon gas only does not affect the expression of ZNRD1 and ZNRD1-AS1. The MCF-7 cells were treated with only argon gas in the two CAP conditions (600 s and 10 × 30 s), and the expression of ZNRD1 (a) and ZNRD1-AS1 (b) was examined by qPCR. All the experiments were performed in triplicate and the values are presented as the mean ± SE. Figure S3: hypermethylation of the CpG at ZNRD1 promoter by CAP treatment to the MCF-7 cells. The effect of CAP on the methylation status of the CpG at the promoter of ZNRD1, which was identified to be hypermethylated from the microarray analysis, was examined by the methylation-specific PCR. All the experiments were performed in triplicate and the values are presented as the mean ± SE. ^∗^*P* < 0.05. Figure S4: optimization of siRNA concentration for the transfection into MCF-7 cells. MCF-7 cells are transiently transfected with a siRNA to induce downregulation of ZNRD1 (a) and ZNRD1-AS1 (b). Downregulation of each gene was judged by qPCR. All the experiments were performed in triplicate and the values are presented as the mean ± SE. ^∗∗^*P* < 0.01, ^∗∗∗^*P* < 0.001. Figure S5: effect of CAP on the expression of ZNRD1 and ZNRD1-AS1 in the T-47D and MCF-10A cells. Expression of ZNRD1 (a) and ZNRD1-AS1 (b) was examined in T-47D and MCF-10A cell line by qPCR after CAP treatment of the cells. All the experiments were performed in triplicate and the values are presented as the mean ± SE. ^∗^*P* < 0.05, ^∗∗^*P* < 0.01, ^∗∗∗^*P* < 0.001.
