# Supplementary material for: ZNRD1 and Its Antisense Long Noncoding RNA ZNRD1-AS1 Are Oppositely Regulated by Cold Atmospheric Plasma in Breast Cancer Cells
Source: Oxid Med Cell Longev. 2020 May 5;2020:9490567. doi: 10.1155/2020/9490567 (PMC7225860; doi:10.1155/2020/9490567)
Supplement: Supplementary Materials — Table S1: specification and electrical characterization of the 90 mm mesh DBD plasma device. Table S2: sequences of primers for qPCR and siRNAs employed in this study. Figure S1: CAP source used in this study. Schematic diagram (a) and voltage-current waveform (b) of the 90 mm mesh DBD plasma device are shown. (c) pH change of culture media after CAP treatment. Figure S2: treatment of argon gas only does not affect the expression of ZNRD1 and ZNRD1-AS1. The MCF-7 cells were treated with only argon gas in the two CAP conditions (600 s and 10 × 30 s), and the expression of ZNRD1 (a) and ZNRD1-AS1 (b) was examined by qPCR. All the experiments were performed in triplicate and the values are presented as the mean ± SE. Figure S3: hypermethylation of the CpG at ZNRD1 promoter by CAP treatment to the MCF-7 cells. The effect of CAP on the methylation status of the CpG at the promoter of ZNRD1, which was identified to be hypermethylated from the microarray analysis, was examined by the methylation-specific PCR. All the experiments were performed in triplicate and the values are presented as the mean ± SE. ∗P < 0.05. Figure S4: optimization of siRNA concentration for the transfection into MCF-7 cells. MCF-7 cells are transiently transfected with a siRNA to induce downregulation of ZNRD1 (a) and ZNRD1-AS1 (b). Downregulation of each gene was judged by qPCR. All the experiments were performed in triplicate and the values are presented as the mean ± SE. ∗∗P < 0.01, ∗∗∗P < 0.001. Figure S5: effect of CAP on the expression of ZNRD1 and ZNRD1-AS1 in the T-47D and MCF-10A cells. Expression of ZNRD1 (a) and ZNRD1-AS1 (b) was examined in T-47D and MCF-10A cell line by qPCR after CAP treatment of the cells. All the experiments were performed in triplicate and the values are presented as the mean ± SE. ∗P < 0.05, ∗∗P < 0.01, ∗∗∗P < 0.001. [file 9490567.f1.docx]

**Supplementary Information**

**ZNRD1 and its anti-sense long non-coding RNA ZNRD1-AS1 are oppositely regulated by cold atmospheric plasma in breast cancer cells**

| **Supplemental Table 1. Specification and electrical characterization of the 90 mm mesh-DBD plasma device.** | |
| --- | --- |
| **Specification** |  |
| Diameter |  |
| Grid | 50 mm |
| Electrode | 50 mm |
| Box nozzle | 50 mm |
| Grid material | Stainless steel |
| Gap between the grid and PLA box | 0.5 mm |
| Glass thickness | 1.8 mm |
| **Electrical characterization** |  |
| Peak voltage | 450 V |
| Discharge voltage* | 280 V |
| Power source | 110 V |
| Gas used | Argon (2 lpm) |
| Voltage (Vrms, kV) | 0.3 |
| Current (Irms, mA) | 12.6 |
| Cycle (us) | 77.6 |
| Frequency (kHz) | 12.9 |
| Plasma discharge energy per cycle (J) | 6.29 x 10^-5^ |
| Energy transfer per second (J/sec) | 8.11 x 10^-1^ |
| *Calculated by averaging values between discharge start and finish voltage. | |

| **Supplemental Table 2. Sequences of primers for qPCR and siRNAs employed in this study.** | | |  |
| --- | --- | --- | --- |
| **Genes** | **Sequence (5'-3') or Product Name** | | **Supplier** |
| **Real-time RT-PC**R | | | |
| *ZNRD1* | TCGATGTGGTCATGAAGGAA | TCCTGGAACTTGCAGTTGGT | Genotech |
| *ZNRD1-AS1* | GATTGGCCTGGGGTACATC | CATCAGAGGACTTGAAATACATGC |  |
| *HLA-A* | TGAGATGGGAGCTGTCTTCC | GCCTGAGTGTAACTCCCTCCT |  |
| *HCG9* | GCCAGGGTGCATCTCAATAA | GAACTCCTGGGCTCAAGCTA |  |
| *PPP1R11* | GCTCATCCAAATGCTGCTGT | TGTGTATGACCACAGCCCTCT |  |
| *TRIM31* | GGGCAGATTCAAGAGCAGAT | ACCTGGTCCGTGAAGACATC |  |
| *RNF39* | TTTGAAGTCCCAACATCCAAG | GTCAGGTCGGCTGTCAGTCT |  |
| *GAPDH* | ACATCGCTCAGACACCATG | TGTAGTTGAGGTCAATGAAGGG | IDT |
| **MSP^a^** | | | |
| *ZNRD1 M* | GTTTTTAGTTGTGTTTGTCG | TACCCCTTCAAAAAAATTTC | Genotech |
| *ZNRD1 U* | GTTTTTAGTTGTGTTTGTTG | TACCCCTTCAAAAAAATTTC |  |
| **siRNA** | | | |
| *siZNRD1-AS1* | FlexiTube GeneSolution for ZNRD1ASP (Cat #. GS80860, SI04958632) | | QIAGEN |
| *siZNRD1* | CAGAAGAGAAUCAGAUCAUtt | AUGAUCUGAUUCUCUUCUGtt | Bioneer |
| *control* | Negative control siRNA (Cat #. SN-1003) | |  |

^a^M, methylated sequence; U, unmethylated sequence.


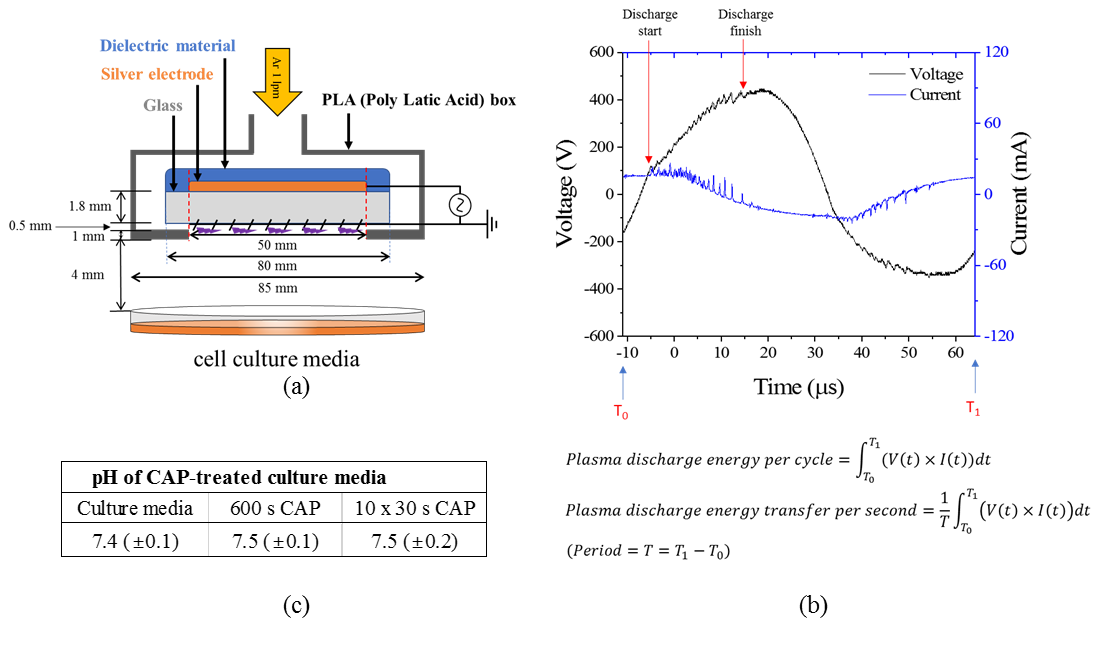
**Supplemental Figure 1**

**
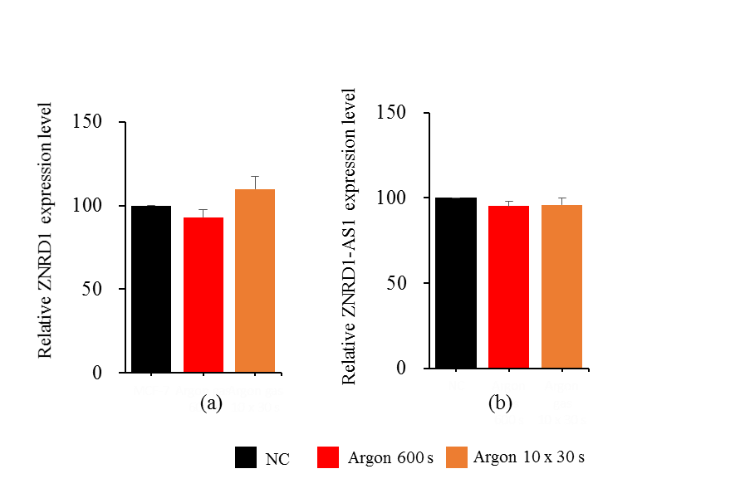
Supplemental Figure 2**

**Supplemental Figure 3**


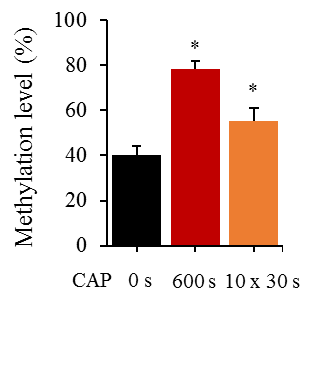


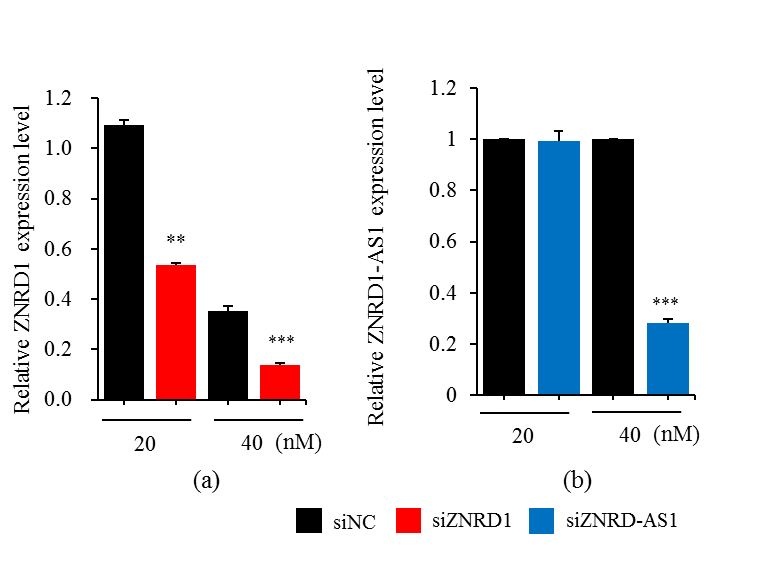
**Supplemental Figure 4**


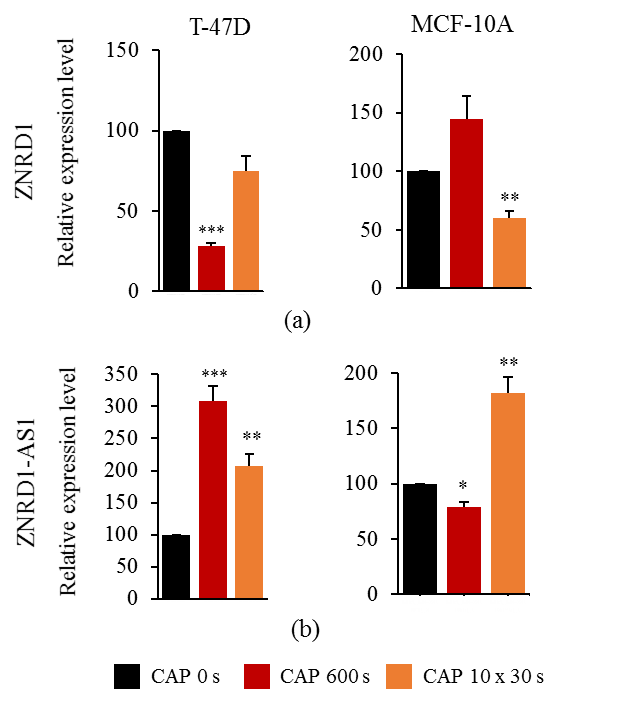
**Supplemental Figure 5**
